# Clinical Remarks by Dr. Marshall Hall, on the Use of Setons

**Published:** 1842-03

**Authors:** 


*Clinical Remarks by* Dr. Marshall Hall, *on the Use of Setons.*—Many years ago, I was consulted by Mr. Doubleday, of Blackfriars Road, in the case of a young married lady who had suffered from peritonitis after her first accouchment.

This peritonitis appeared to be confined to the pelvic region. Its acute character had been subdued, but tenderness, with tumidity, and difficulty in voiding the bladder and rectum remained. I made a careful examination. A distinct hardness was felt under the pubes, extending to one side, I think the left. On examination per vaginam and per rectum, a similar hardness was found occupying the lower part of the pelvis. I imagined this hardness to consist in coagulable lymph, effused from the inflamed peritoneal surfaces of the pelvis, producing the symptoms by its pressure on the neck of the bladder and on the rectum.

We strictly regulated the diet and the intestines, and inserted an ample seton over the induration. Slowly and gradually that induration, with its attendant symptoms, became diminished, and eventually disappeared.

Several years after this, I was consulted in the case of the sister-in-law of this patient, under very nearly similar circumstances. The same remedy was followed by the same happy result.

A year ago I was consulted by Mr. Burford, in the case of a gentleman of sixty, who had become affected with pain, tenderness, and tumidity of the abdomen. On a careful examination, a distinct hardness was felt in the midst of the general tumidity, occupying the region of the caput cœcum coli. We regulated the diet and the bowels, administered mercury, and inserted an ample seton. The mouth became affected, and the seton discharged copiously: the hardness and the other symptoms gradually, but at length entirely disappeared

A similar case occurred a year ago, in the person of a gentleman of forty, a patient of Mr. Squibb, in Orchard street. A strict regimen was enjoined, the bowels regulated, and an ample seton was inserted. The induration, which in this case occupied the space between the false ribs and the ilium, on the left side, gradually disappeared.

Two years ago I was consulted by Mr. P———, a barrister, affected with pneumonia of the middle and upper lobes of the right lung. A seton was inserted, and Mr. P——— went to Madeira. On his return, the physical signs and the symptoms of pneumonia had disappeared.

I have still more recently treated a case of pneumonia of the upper part of the right lung, in consultation with Mr. Beane of Peckham. A seton was inserted, and in six weeks a most decided amendment in the physical signs, the symptoms, and the general health occurred. Since that period the patient has continued to improve, and now no dullness on percussion, or other signs of disease, is perceptible.

In a variety of cases of acute or chronic, local or limited internal inflammation, I have had recourse to the seton, and uniformly with the most marked success; so that, I think, we may look upon the remedy as almost specific in such cases. It is unnecessary to enumerate them. But hepatitis and nephritis belong to them in an especial manner, and I would suggest this remedy as likely to be of service (if any remedy can) in the case of albuminous urine. In one such case the urine was more albuminous after cupping. I imagined the effect arose from the mechanical violence inflicted, and recommended the cupping to be performed above and below the precise region of the kidneys. Under the use of this remedy the albumen diminished, and even ceased for a time.

These and other cases, then, induce me to think that there can be little doubt of the real efficacy of the seton in chronic inflammation. The object is to demonstrate this in some measure, and then to notice briefly farther applications of the remedy. I do not pretend to suggest any thing new, but rather to enforce what is old. The efficacy of setons, when appropriately applied in the nucha (for they are frequently employed very uselessly), is well known. The proper cases are inflammation and congestion. But the case to which I would particularly draw attention is that of disease of the spinal marrow, with paraplegia, or paraplegic spasm.

In this case issues are generally inserted. They appear to me far more painful, far less manageable, and far less efficacious than ample setons. They have also, I am persuaded, been generally applied below the real seat of the disease. I was consulted a few weeks ago by a gentleman from Manchester. With partial loss of power, he had loss of sensation in the lower extremities; the numbness extended to a line just above the sacrum. Issues had been applied on each side of this line. They might, with equal efficacy, have been applied to the foot! I need not say that the spinal nerves proceed, for some distance, from above directly, rather than obliquely, downwards, and that the seat of the disease is *at* or ABOVE their junction (insertion or origin) with the spinal marrow.

Bearing these two principles in mind, then, viz. that ample setons afford a more efficacious counter irritation than issues, and that they ought to be applied higher along the spinal column than has been usual, I think we have a new mode of treatment for this formidable class of diseases.

These setons should, besides, be larger than usual. They should be three-fourths of an inch in breadth, and extend through two inches in length, be inserted on the level with and above the supposed seat of the disease (the anatomy being consulted), and be four or six in number, two or three being instituted on each side of the spinal column. Acting on this principle, I had, five days ago, the pleasure to receive the most satifactory account of a patient affected with paraplegia whom I had seen at Lohan, in Essex, in consultation with Mr. Gross.

I repeat, and beg to conclude by repeating, that I believe counter irritation applied along the spine has failed, because it has been applied below the seat of the disease; and that, to be efficacious, it must be both more efficient in itself, and applied with greater regard to the anatomy of the spinal marrow and nerves. The precise spot for their application must be left to the well-informed practitioner. I need scarcely remind my reader, that the persistence or cessation of all reflex actions, will determine whether our remedies should be applied above or below the origin of the cauda equina, above or below the last dorsal vertebra.

We are great advocates for setons too. We confess we have not used them so broad nor so many at a time as our friend Dr. Hall. But perhaps he is right. The difficulty would be, in some cases, to induce patients to submit to their introduction.

We have tried setons in three or four cases of albuminous urine. They were bad cases certainly, and the effect was not encouraging.—*London Medico-Chirurgical Review*, from *London and Edinburgh Journal of Med. Science, No.* 11.

